# Thermoresponsive and Protease‐Cleavable Interferon‐Polypeptide Conjugates with Spatiotemporally Programmed Two‐Step Release Kinetics for Tumor Therapy

**DOI:** 10.1002/advs.201900586

**Published:** 2019-06-14

**Authors:** Zhuoran Wang, Jianwen Guo, Jiawei Sun, Ping Liang, Yan Wei, Xuliang Deng, Weiping Gao

**Affiliations:** ^1^ Department of Biomedical Engineering School of Medicine Tsinghua University Beijing 100084 P. R. China; ^2^ Department of Neurosurgery Beijing Tsinghua Changgung Hospital School of Clinical Medicine Tsinghua University Beijing 102218 P. R. China; ^3^ Department of Geriatric Dentistry Beijing Laboratory of Biomedical Materials Peking University School and Hospital of Stomatology Beijing 100081 P.R. China; ^4^ Department of Geriatric Dentistry Beijing Laboratory of Biomedical Materials Peking University School and Hospital of Stomatology Beijing 100081 P. R. China; ^5^ Biomedical Engineering Department Peking University Beijing 100191 P. R. China

**Keywords:** elastin‐like polypeptides, interferon, matrix metalloproteinases, protein‐polymer conjugates, tumor microenvironment

## Abstract

Protein‐polymer conjugates show improved pharmacokinetics but reduced bioactivity and tumor penetration as compared to native proteins, resulting in limited antitumor efficacy. To address this dilemma, genetic engineering of a body temperature‐responsive and matrix metalloproteinase (MMP)‐cleavable conjugate of interferon alpha (IFNα) and elastin‐like polypeptide (ELP) is reported with spatiotemporally programmed two‐step release kinetics for tumor therapy. Notably, the conjugate could phase separate to form a depot postsubcutaneous injection, leading to 1‐month zero‐order release kinetics. Furthermore, it could selectively be cleaved by MMPs that are overexpressed in tumors to release IFNα from ELP and thus to recover the bioactivity of IFNα. Consequently, it exhibits dramatically enhanced tumor accumulation, tumor penetration, and antitumor efficacy as compared to free IFNα in two mouse models of melanoma and ovarian tumor. These findings may provide an intelligent technology of thermoresponsive and protease‐cleavable protein‐polymer conjugates with spatiotemporally programmed two‐step release kinetics for tumor treatment.

## 1 Introduction

Protein therapeutics are increasingly important for the treatments of various diseases, such as cancer, diabetes, infectious, and inflammatory diseases.^1^, ^2^ However, most protein therapeutics have short circulatory half‐lives owing to their rapid renal clearance and poor stability.^3^, ^4^ As a result, they need to be frequently injected at high dosages, leading to not only high treatment cost and poor patient compliance but also low therapeutic efficacy and severe side effects. Conjugation of a protein‐resistant polymer, typically poly(ethylene glycol) (PEG), to a protein to form protein‐polymer conjugates is a frequently used method to elongate the half‐life of the protein.^5^, ^6^, ^7^, ^8^, ^9^, ^10^ Indeed, 16 PEGylated protein therapeutics have been clinically used in the world.^10^ Nevertheless, although PEGylation can substantially enhance the pharmacokinetics of proteins, it can reduce the bioactivity of the proteins considerably.^7^, ^11^ For instance, the half‐life of PEGASYS (49.5 h), a food and drug administration‐approved PEGylated interferon alpha (IFNα), is 61.9‐fold longer than that of free IFNα (0.8 h), whereas the bioactivity of PEGASYS is 51.3‐fold lower than that of free IFNα.^11^ On the other hand, the enlarged size caused by polymer conjugation of protein can reduce tumor penetration of the protein. The compromise between improved pharmacokinetics and reduced bioactivity and tumor penetration limits the antitumor efficacy of protein‐polymer conjugates.

**Scheme 1:**
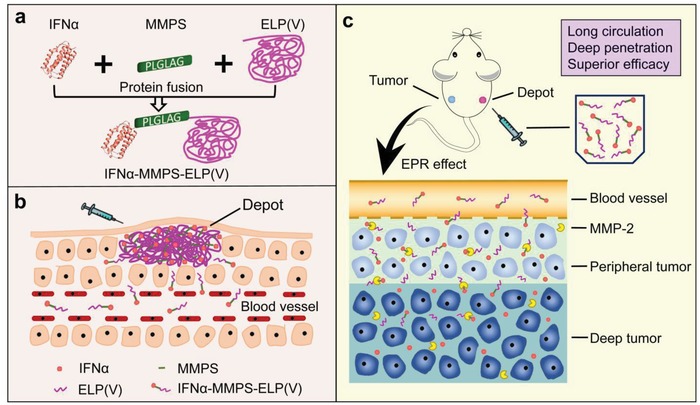
a) Genetic engineering of a body temperature‐responsive and MMP‐cleavable protein‐polymer conjugate of IFNα‐MMPS‐ELP(V) with spatiotemporally programmed two‐step release kinetics for improved tumor therapy. b) The conjugate can generate a depot postsubcutaneous injection and slowly liberate from the depot into circulatory system for 1 month. c) The released conjugate can get into a tumor through the EPR effect and then be cleaved into free IFNα and ELP(V) by MMP‐2 in the tumor, resulting in enhanced tumor penetration and antitumor efficacy.

To address this dilemma, we introduce stimuli‐responsive functions into protein‐polymer conjugates to design body temperature‐responsive and matrix metalloproteinase (MMP)‐cleavable protein‐polymer conjugates with spatiotemporally‐programed two‐step release kinetics for enhanced tumor therapy. In this proof‐of‐concept study, a MMP substrate (MMPS) as a linker was genetically fused to the C‐terminal of IFNα and the N‐terminal of a body temperature‐responsive elastin‐like polypeptide (ELP(V)) to form a body temperature‐responsive and MMP‐cleavable IFNα‐MMPS‐ELP(V) conjugate (**Scheme** 1a). IFNα is clinically used for the treatments of hepatitis and cancer. Its clinically relevant activity has been ascribed to its immunostimulatory functions although it was initially thought to directly kill cancer cells by activating IFNα receptor signaling in cancer cells.^12^ ELPs are a kind of biodegradable, biocompatible, and thermoresponsive biopolymers comprised of Val‐Pro‐Gly‐Xaa‐Gly repeat unit in which Xaa is the guest residue.^13^, ^14^, ^15^ ELPs have increasingly been used as carriers for the delivery of small molecule drugs,^15^, ^16^, ^17^, ^18^, ^19^, ^20^ peptide and protein therapeutics,^21^, ^22^, ^23^, ^24^, ^25^, ^26^ and nanoparticles.^27^, ^28^ MMPs such as MMP‐2 and MMP‐9 are overexpressed in tumors to degrade extracellular matrix, allowing cancer cells to migrate from the primary tumor to form metastases.^29^ This pathophysiological phenomenon has been utilized in drug delivery systems to release drugs from the carriers in tumors.^30^, ^31^, ^32^, ^33^ On the basis of these principles, we reason that the IFNα‐MMPS‐ELP(V) conjugate would phase separate to form a depot postsubcutaneous injection and slowly liberate from the depot into circulatory system due to the concentration dependence of the phase transition temperature (*T*_t_) of the conjugate, resulting in dramatically improved pharmacokinetics (Scheme 1b). Furthermore, the released conjugate would accumulate into tumors through the enhanced permeation and retention effect (EPR).^34^ It would be cleaved into free IFNα and ELP(V) by MMPs in the tumors, leading to not only the recovery of IFNα bioactivity but also the enhancements in tumor penetration and antitumor efficacy (Scheme 1c). Indeed, in two mouse models of melanoma and ovarian tumor, the conjugate showed remarkably increased tumor accumulation, tumor penetration, and antitumor efficacy as well as dramatically enhanced pharmacokinetics as compared to free IFNα.

## 2 Results and Discussion

### 2.1 Synthesis and Physicochemical and Bioactive Characterizations

**Figure 1:**
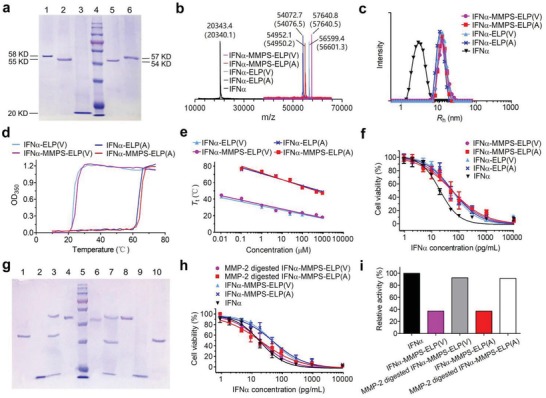
In vitro characterization of IFNα‐MMPS‐ELP(V). a) SDS‐PAGE analyses after purification. Lane 1: IFNα‐MMPS‐ELP(V), lane 2: IFNα‐MMPS‐ELP(A), lane 3: IFNα, lane 4: marker, lane 5: IFNα‐ELP(A), lane 6: IFNα‐ELP(V). b) MALDI‐TOF‐MS spectra. The theoretical mass of each sample is shown in parenthesis. c) DLS analyses in which *R*_h_ is hydrodynamic radius. d) Turbidity (OD_350_) versus temperature at 25 × 10^−6^ m in phosphate buffer saline (PBS). e) The concentration dependence of *T*_t_. f) In vitro cytotoxicity to Daudi B cells. g) SDS‐PAGE analyses after incubation with MMP‐2. Lane 1: ELP(V) (37 kD), lanes 2 and 9: IFNα (20 kD), lane 3: IFNα‐MMPS‐ELP(V) incubated with MMP‐2 (MMP‐2 62 kD, ELP(V) 37 kD, IFNα 20 kD), lane 4: IFNα‐MMPS‐ELP(V) (58 kD), lane 5: marker, lane 6: IFNα‐MMPS‐ELP(A) (55 kD), lane 7: IFNα‐MMPS‐ELP(A) incubated with MMP‐2 (MMP‐2 62 kD, ELP(A) 35 kD, IFNα 20 kD), lane 8: MMP‐2 (62 kD), and lane 10: ELP(A) (35 kD). h) In vitro cytotoxicity to Daudi B cells after MMP‐2 treatments. i) The relative antiproliferative activities after MMP‐2 treatments as compared to that of IFNα.

In this study, two IFNα‐MMPS‐ELP conjugates of IFNα‐MMPS‐ELP(V) and IFNα‐MMPS‐ELP(A) with distinct *T*_t_ values were genetically engineered (see Supporting Information). Simultaneously, two MMP‐noncleavable IFNα‐ELP conjugates of IFNα‐ELP(V) and IFNα‐ELP(A) with distinct *T*_t_ values were prepared as controls. Specifically, the sequence of MMPS was Pro‐Leu‐Gly‐Leu‐Ala‐Gly (PLGLAG). The A and V in ELP(A) and ELP(V) were the X in VPGXG repeat unit. The repeat unit number per ELP chain was 90. The conjugates were analyzed by sodium dodecyl sulfate polyacrylamide gel electrophoresis (SDS‐PAGE) and further confirmed by matrix‐assisted laser desorption/ionization time‐of‐flight mass spectrometry (MALDI‐TOF‐MS) to have the expected molecular weights (**Figure** 1a,b). The hydrodynamic radii (*R*_h_) of the conjugates were determined by dynamic light scattering (DLS) to be around 11 nm, which were 3.8‐fold larger than that of IFNα (2.9 nm) (Figure 1c). The circular dichroism (CD) spectra of the conjugates were almost identical to that of IFNα (Figure S1, Supporting Information), indicating that the ELP conjugation of IFNα did not change the secondary structure of IFNα. The thermoresponsive phase transition behaviors of the conjugates were studied by turbidity measurement (Figure S2, Supporting Information). The conjugates showed sharp phase transitions with concentration‐dependent *T*_t_ (Figure 1d,e). The *T*_t_ values of IFNα‐MMPS‐ELP(V) and IFNα‐ELP(V) were below the body temperature of 37 °C when the concentrations were above 1 × 10^−6^ m. These data suggested that IFNα‐MMPS‐ELP(V) and IFNα‐ELP(V) would in situ form depots upon subcutaneous injections at high concentrations and then would slowly liberate from the depots into circulatory system at low concentrations. By contrast, the *T*_t_ values of IFNα‐MMPS‐ELP(A) and IFNα‐ELP(A) were well above the body temperature even if the concentrations were as high as 1000 × 10^−6^ m, suggesting that they could not in situ form depots upon subcutaneous injections. The antiproliferative activities of the conjugates (half maximal inhibitory concentration, IC_50_ = 55.3 pg mL^−1^ for IFNα‐MMPS‐ELP(V), 53.8 pg mL^−1^ for IFNα‐MMPS‐ELP(A), 54.6 pg mL^−1^ for IFNα‐ELP(V), 56.9 pg mL^−1^ for IFNα‐ELP(A)) were nearly the same, which were around 37% of that of IFNα (IC_50_ = 20.2 pg mL^−1^) (Figure 1f), indicating that these conjugates had the same antiproliferative activity because of the same ELP chain length.

### 2.2 The MMP‐Cleavable Property of IFNα‐MMPS‐ELP In Vitro

To study the MMP‐cleavable property of IFNα‐MMPS‐ELP(V) and IFNα‐MMPS‐ELP(A), the conjugates were treated with MMP‐2, followed by SDS‐PAGE analysis (Figure 1g). As expected, the conjugates were cleaved into two species of IFNα and ELP, while IFNα‐ELP(V) and IFNα‐ELP(A) could not be cleaved by MMP‐2 (Figure S3, Supporting Information). These results were further confirmed by incubating the conjugates with ovarian tumor cells and melanoma cells conditioned media (Figure S4, Supporting Information), which revealed that IFNα‐MMPS‐ELP(V) and IFNα‐MMPS‐ELP(A) could be cleaved into IFNα and ELP by MMPs secreted by the tumor cells. Notably, after the MMP‐2 treatment, the antiproliferative activities of IFNα‐MMPS‐ELP(V) (21.8 pg mL^−1^, IC_50_) and IFNα‐MMPS‐ELP(A) (22.1 pg mL^−1^, IC_50_) were increased from 37% to about 91% of that of IFNα (20.2 pg mL^−1^, IC_50_) (Figure 1h,i), while the bioactivities of IFNα‐ELP(A) and IFNα‐ELP(V) did not change (Figure S5, Supporting Information), as expected. These data indicated that IFNα‐MMPS‐ELP(V) and IFNα‐MMPS‐ELP(A) could be cleaved by MMP‐2 to release IFNα from ELP and thus recover the bioactivity to a high degree.

### 2.3 The Effect of the MMP‐Cleavable Function of IFNα‐MMPS‐ELP(A) on Its In Vivo Properties

**Figure 2:**
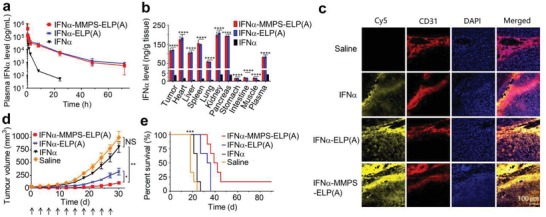
The effect of the MMP‐cleavable function of IFNα‐MMPS‐ELP(A) on its in vivo properties in a melanoma mouse model. a) Plasma IFNα level versus time postintravenous injection at a dosage of 1 mg IFNα‐equivalent/kg BW (*n* = 3). b) IFNα levels in the tumors and major organs 8 h postadministration (*n* = 3, ****P < 0.0001, substantial difference for IFNα‐MMPS‐ELP(A) versus IFNα and IFNα‐ELP(A)). c) Tumor penetration. The cell nucleus is in blue after stained with 4', 6‐diamidino‐2‐phenylindole (DAPI); the vessels are in red after stained with rat antimouse CD31 antibody as the first antibody and finally labeled with cyanine 3 (Cy3); IFNα, IFNα‐ELP(A), and IFNα‐MMPS‐ELP(A) tagged with Cy5 are in yellow. d) Inhibition of melanoma growth postintravenous injection at a dose of 1.5 mg IFNα‐equivalent per kg BW (*n* = 8–10, *P < 0.05, **P < 0.01). The arrows denote the time points of dosing. e) Survival of the mice bearing melanoma post intravenous injection (*n* = 8–10, ***P < 0.001, substantial difference for IFNα‐MMPS‐ELP(A) compared to IFNα and IFNα‐ELP(A)).

We further studied the effect of the MMP‐cleavable function of IFNα‐MMPS‐ELP(A) on its in vivo properties in a mouse model of C8161 melanoma (**Figure** 2). The pharmacokinetics of IFNα‐MMPS‐ELP(A) was similar to that of IFNα‐ELP(A) but much better than that of free IFNα (Figure 2a and Table S1, Supporting Information). For instance, the half‐lives (*t*_1/2_) of IFNα‐MMPS‐ELP(A) (8.9 ± 1.0 h) and IFNα‐ELP(A) (9.6 ± 2.7 h) were 6.4‐ and 6.9‐fold longer than that of free IFNα (1.4 ± 0.21 h), respectively. The area under the curves (AUCs) of IFNα‐MMPS‐ELP(A) (684.1 ± 12.1 µg L^−1^·h) and IFNα‐ELP(A) (707.3 ± 37.1 µg L^−1^ h) were 11.1‐ and 11.4‐fold larger than that of free IFNα (61.8 ± 1.7 µg L^−1^·h), respectively. Similarly, the biodistribution of IFNα‐MMPS‐ELP(A) was almost the same as that of IFNα‐ELP(A) but much superior to that of free IFNα (Figure 2b). Notably, the tumor concentrations of IFNα‐MMPS‐ELP(A) (117.7 ng IFNα‐equivalent per g tissue) and IFNα‐ELP(A) (120.8 ng IFNα‐equivalent per g tissue) were 32.7‐ and 33.6‐fold higher than that of free IFNα (3.6 ng g^−1^ tissue), respectively. These data indicated that the introduction of the MMPS linker into IFNα‐ELP(A) did not change the pharmacokinetics and biodistribution significantly. However, IFNα‐MMPS‐ELP(A) showed significantly enhanced tumor penetration as compared to both IFNα‐ELP(A) and free IFNα, as indicated by the more intense fluorescence (yellow) observed in the area that was distant from the vessels (red) for IFNα‐MMPS‐ELP(A) than for IFNα‐ELP(A) and free IFNα (Figure 2c and Figure S6, Supporting Information). This phenomenon could be ascribed to the cleavage of IFNα‐MMPS‐ELP(A) by MMP‐2 overexpressed in the tumor into IFNα and ELP(A). As a result, IFNα‐MMPS‐ELP(A) more efficiently suppressed tumor growth than IFNα‐ELP(A) and free IFNα (Figure 2d and Figure S7, Supporting Information). The median survival time for IFNα‐MMPS‐ELP(A) (40.5 d) was 1.2‐, 1.7‐, and 2.3‐fold longer than those for IFNα‐ELP(A) (34.5 d), free IFNα (24 d), and saline (18 d), respectively (Figure 2e). Notably, 16.7% of the mice in the IFNα‐MMPS‐ELP(A) treatment group were tumor free. No significant loss in body weight (BW) was found for all of the treatments (Figure S8, Supporting Information). Taken together, all the results indicated that the introduction of the MMPS linker into IFNα‐ELP(A) did not change the pharmacokinetics and biodistribution but improved the tumor penetration and antitumor efficacy.

### 2.4 Pharmacokinetics, Biodistribution, and Tumor Penetration of IFNα‐MMPS‐ELP(V)

**Figure 3:**
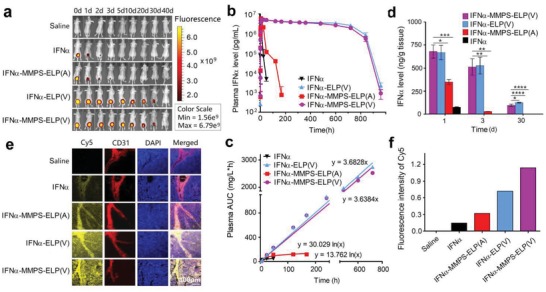
Pharmacokinetics, biodistribution, and tumor penetration of IFNα‐MMPS‐ELP(V) after subcutaneous administration at its MTD in a melanoma mouse model. a) Fluorescence imaging at the indicated time points after subcutaneous injections of Cy5 labeled conjugates at their MTDs. b) Plasma IFNα level versus time postsubcutaneous administration at its MTD (*n* = 3). c) AUC versus time (*n* = 3). d) IFNα levels in the tumors 1, 3, and 30 d postdosing (*n* = 3, *P < 0.05, **P < 0.01, ***P < 0.001, ****P < 0.0001, substantial difference for IFNα‐MMPS‐ELP(V) versus IFNα‐MMPS‐ELP(A), IFNα‐ELP(V), and IFNα). e) Tumor penetration. The cell nucleus is in blue after stained with DAPI; the vessels are in red after stained with rat antimouse CD31 antibody as the first antibody and finally labeled with Cy3; IFNα‐MMPS‐ELP(V), IFNα‐MMPS‐ELP(A), IFNα‐ELP(V), and IFNα tagged with Cy5 are in yellow. f) The quantitative analysis of Cy5 fluorescence intensity is shown in panel (e).

On the basis of these in vivo findings of IFNα‐MMPS‐ELP(A), we further studied the in vivo properties of IFNα‐MMPS‐ELP(V) postsubcutaneous administration at its maximum tolerated dose (MTD). The MTD (100 mg kg^−1^ body weight) for IFNα‐MMPS‐ELP(V) was the same as that for IFNα‐ELP(V), which was 5.0‐ and 6.7‐fold higher than those for IFNα‐MMPS‐ELP(A) (20 mg kg^−1^ body weight) and free IFNα (15 mg kg^−1^ body weight), respectively (Figure S9, Supporting Information). Interestingly, a bump in situ formed and gradually diminished over 40 d after subcutaneous injection of IFNα‐MMPS‐ELP(V) or IFNα‐ELP(V) at its MTD (Figure S10, Supporting Information). This phenomenon suggested that both IFNα‐MMPS‐ELP(V) and IFNα‐ELP(V) could in situ form depots after subcutaneous injections at their MTDs because the *T*_t_ values of the two conjugates were far below the in vivo temperature (Figure 1e). Meanwhile, they could gradually liberate from the depots into circulatory system due to the concentration dependence of *T*_t_ (Figure 1e). In contrast, IFNα‐MMPS‐ELP(A) could not generate a depot postsubcutaneous injection at its MTD because the *T*_t_ value at its MTD was well above the body temperature (Figure 1e). These results were further confirmed by fluorescence imaging after subcutaneous injections of cyanine 5 (Cy5) labeled conjugates at their MTDs, as indicated by the observation that the fluorescence intensity of IFNα‐MMPS‐ELP(V) and IFNα‐ELP(V) gradually diminished over 40 d, while the fluorescence intensity of IFNα‐MMPS‐ELP(A) quickly dropped within 5 d (**Figure** 3a). The pharmacokinetics of IFNα‐MMPS‐ELP(V) was studied after subcutaneous administration at the MTD (Figure 3b). The plasma IFNα levels of IFNα‐MMPS‐ELP(V) and IFNα‐ELP(V) increased to 5416.9 ± 589.3 and 5461.1 ± 558.8 µg L^−1^ at 13.2 h, respectively, almost remained persistent until day 30, and then started to decrease, showing 1‐month zero‐order controlled release. By contrast, the plasma IFNα levels of IFNα‐MMPS‐ELP(A) and IFNα rapidly increased to 5878.7 ± 187.1 µg L^−1^ at 4.0 h and 5456.9 ± 758.5 µg L^−1^ at 2.8 h, respectively, and then decreased quickly. Representative pharmacokinetic parameters were generated by fitting the data with a one‐compartment model (Table S2, Supporting Information). Notably, The *t*_1/2_ of IFNα‐MMPS‐ELP(V) (422.2 ± 13.7 h) was close to that of IFNα‐ELP(V) (491.8 ± 38.1 h), but was 46.9‐ and 222.2‐fold longer than those for IFNα‐MMPS‐ELP(A) (9.0 ± 0.87 h) and free IFNα (1.9 ± 0.08 h), respectively. The AUC of IFNα‐MMPS‐ELP(V) (2755.9 ± 16.8 mg L^−1^·h) was close to that of IFNα‐ELP(V) (3102.0 ± 269.8 mg L^−1^·h), but was 23.3‐ and 58.8‐fold larger than those for IFNα‐MMPS‐ELP(A) (118.3 ± 15.1 mg L^−1^ h) and free IFNα (46.9 ± 7.8 mg L^−1^·h), respectively. Furthermore, the AUCs of IFNα‐MMPS‐ELP(V) and IFNα‐ELP(V) were correlated to time linearly (Figure 3c), indicating the zero‐order sustained release for 1 month, whereas the AUCs of IFNα‐MMPS‐ELP(A) and IFNα were logarithmically correlated to time. These data showed that the introduction of the MMPS linker into IFNα‐ELP(V) did not significantly change the dramatically improved pharmacokinetics and tolerability as compared to IFNα‐MMPS‐ELP(A) and IFNα.

Next, the biodistribution of IFNα‐MMPS‐ELP(V) was studied after subcutaneous administration at the MTD. The biodistribution of IFNα‐MMPS‐ELP(V) was similar to that of IFNα‐ELP(V), but was much better than those of IFNα‐MMPS‐ELP(A) and free IFNα (Figure S11, Supporting Information). Notably, the tumor concentration of IFNα‐MMPS‐ELP(V) (539.3 ± 165.5 ng g^−1^ tissue) was nearly equal to that of IFNα‐ELP(V) (555.7 ± 164.7 ng g^−1^ tissue), but was 18.0‐ and 317.2‐fold higher than those for IFNα‐MMPS‐ELP(A) (29.9 ± 1.8 ng g^−1^ tissue) and free IFNα (1.7 ± 0.05 ng g^−1^ tissue) at 3 d postinjection, respectively (Figure 3d). As expected, the tumor penetration of IFNα‐MMPS‐ELP(V) was much better than those of IFNα‐ELP(V), IFNα‐MMPS‐ELP(A), and free IFNα, as indicated by the more intense fluorescence (yellow) in the area that was distant from the vessels (red) for IFNα‐MMPS‐ELP(V) than for the controls (Figure 3e,f). Collectively, all of the data indicated that IFNα‐MMPS‐ELP(V) had remarkably improved MTD, pharmacokinetics, and tumor accumulation as compared to IFNα‐MMPS‐ELP(A) and free IFNα mainly due to the body temperature‐responsive controlled release. Although IFNα‐MMPS‐ELP(V) was close to IFNα‐ELP(V) in these properties, it had enhanced tumor penetration over IFNα‐ELP(V) due to the MMP‐cleavable function, which suggested that IFNα‐MMPS‐ELP(V) would have better antitumor efficacy than IFNα‐ELP(V).

### 2.5 Antitumor Efficacy of IFNα‐MMPS‐ELP(V)

**Figure 4:**
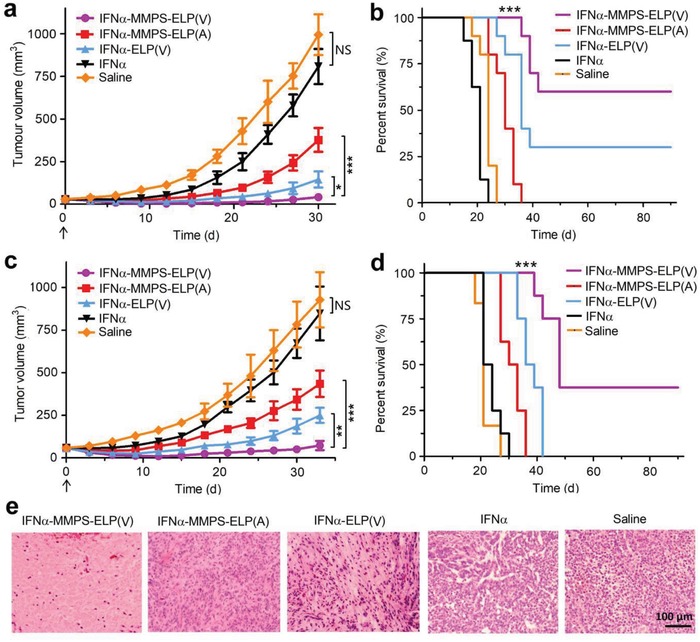
Antitumor efficacy of IFNα‐MMPS‐ELP(V) postsubcutaneous injection at its MTD in melanoma and ovarian mouse models. a) Melanoma growth inhibition (*n* = 8–10, *P < 0.05, ***P < 0.001). The arrow denotes the time point of dosing. b) Survival of the animals bearing melanomas (*n* = 8–10, ***P < 0.001, substantial difference for IFNα‐MMPS‐ELP(V) versus IFNα‐MMPS‐ELP(A), IFNα‐ELP(V), IFNα, and saline). c) Inhibition of ovarian tumor growth (*n* = 6–8, **P < 0.01, ***P < 0.001). The arrow denotes the time point of dosing. d) Survival of the animals bearing ovarian tumors (*n* = 6–8, ***P < 0.001, substantial difference for IFNα‐MMPS‐ELP(V) versus IFNα‐MMPS‐ELP(A), IFNα‐ELP(V), IFNα, and saline). e) Tumor tissue H&E staining after subcutaneous injection.

To certify the hypothesis, we first evaluated the antitumor efficacy of IFNα‐MMPS‐ELP(V) in a mouse model of melanoma. After subcutaneous injection at its MTD, IFNα‐MMPS‐ELP(V) suppressed melanoma growth more efficiently than IFNα‐ELP(V), IFNα‐MMPS‐ELP(A), and free IFNα (**Figure** 4a). The average tumor size for IFNα‐MMPS‐ELP(V) (41.5 ± 11.4 mm^3^) was 3.5‐, 9.1‐, and 19.5‐fold smaller than those for IFNα‐ELP(V) (145.3 ± 49.1 mm^3^), IFNα‐MMPS‐ELP(A) (376.8 ± 121.7 mm^3^), and free IFNα (808.2 ± 226.1 mm^3^) at 30 d after the subcutaneous injections, respectively. Notably, 60% of the mice in the IFNα‐MMPS‐ELP(V) treatment group were tumor free, which was much higher than those for the treatments with IFNα‐ELP(V) (30%), IFNα‐MMPS‐ELP(A) (0%), and free IFNα (0%) (Figure 4b). Furthermore, the improvement in inhibiting tumor growth of IFNα‐MMPS‐ELP(V) over the controls was also observed in another mouse model of ovarian tumor (Figure 4c). The average tumor size for IFNα‐MMPS‐ELP(V) (72.9 ± 17.8 mm^3^) was 3.4‐, 6.0‐, and 11.6‐fold smaller than those for IFNα‐ELP(V) (250.4 ± 24.9 mm^3^), IFNα‐MMPS‐ELP(A) (434.9 ± 119.9 mm^3^), and free IFNα (847.9 ± 248.2 mm^3^) at 33 d after the subcutaneous injections, respectively. Notably, 37.5% of the mice in the IFNα‐MMPS‐ELP(V) treatment group were tumor free, whereas the tumor cure percentages for the treatments with IFNα‐ELP(V), IFNα‐MMPS‐ELP(A), and free IFNα were 0% (Figure 4d). The enhanced antimelanoma efficacy was further confirmed by hematoxylin‐eosin (H&E) staining, as indicated by a more extensive degree of destruction observed for IFNα‐MMPS‐ELP(V) than for the controls (Figure 4e). Taken together, these results demonstrated that IFNα‐MMPS‐ELP(V) was much more efficient in antitumor efficacy than the controls due to the synergy of the body temperature‐responsive and MMP‐cleavable functions.

### 2.6 Biosafety of IFNα‐MMPS‐ELP(V)

**Figure 5:**
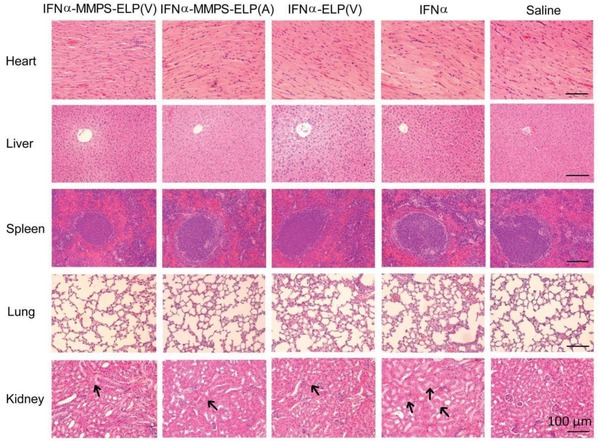
H&E staining of major tissues of mice at 12 d after subcutaneous injections of IFNα‐MMPS‐ELP(V), IFNα‐MMPS‐ELP(A), IFNα‐ELP(V), and IFNα at their MTDs in a melanoma mouse model. In the figure of kidney, the arrows indicate the tissue damage sites.

All the treatments did not cause obvious loss of body weight (Figure S12, Supporting Information). As indicated by hematoxylin and eosin (H&E) staining, a more extensive degree of kidney damage was observed in the IFNα treatment than in the other treatments, but no histological change in other major organs was observed in all the treatments (**Figure** 5). This result was further validated by blood biochemistry analysis, which revealed that the levels of markers for kidney function in the IFNα treatment were much higher than those in the other treatments (Figure S13, Supporting Information). Additionally, no significant change in the levels of important hematological markers was observed in all the treatments (Figure S14, Supporting Information). All the results indicated that IFNα‐MMPS‐ELP(V) could reduce the systemic toxicity of IFNα.

## 3 Conclusion

In conclusion, we have reported genetic engineering of a body‐temperature‐responsive and MMP‐cleavable protein‐polymer conjugate of IFNα‐MMPS‐ELP(V) with not only dramatically improved pharmacokinetics but also remarkably enhanced tumor accumulation and penetration, and antitumor efficacy. IFNα‐MMPS‐ELP(V) can be genetically engineered to own dual functions of body temperature responsiveness and MMP cleavability that enable spatiotemporally programmed two‐step release kinetics. The body temperature responsiveness can remarkably enhance the tolerability, pharmacokinetics, and tumor accumulation of the conjugate through the mechanism of body temperature‐responsive controlled release due to the concentration dependence of the phase transition temperature. Notably, in a mouse model, subcutaneous injection of the conjugate at its MTD offers an extremely prolonged circulation half‐life of 422.2 h. The half‐life of the conjugate in human is expected to be as long as 1195.5 h according to the empirical formulus of *t*_human_ = *t*_mouse_ (*W*_human_/*W*_mouse_)^0.13^ in which *t*_human_ and *t*_mouse_ are the half‐lives of the conjugate in human and mouse, respectively, and *W*_human_ and *W*_human_ are the body weights of human and mouse, respectively.^35^, ^36^, ^37^ This result suggests that the conjugate might be administered at a frequency of once every 3 months in humans. The super‐long half‐life is highly beneficial to patients in reducing side effects and improving patient compliance. The MMP cleavability makes it possible to release IFNα from ELP(V) to not only recover the bioactivity but also enhance the tumor penetration when the conjugate accumulates into a tumor through the EPR effect. The synergy of the body temperature responsiveness and MMP cleavability leads to the dramatically improved antitumor efficacy while decreased side effects as compared to free IFNα in two mouse models of melanoma and ovarian tumor. These findings implicate that protein‐polymer conjugates with thermoresponsiveness and protease cleavability would be a next‐generation technology to dramatically enhance the antitumor efficacy of therapeutic proteins while reducing side effects.

## Conflict of Interest

The authors declare no conflict of interest.

## Supporting information

Supplementary
