# Supplementary material for: Thermoresponsive and Protease‐Cleavable Interferon‐Polypeptide Conjugates with Spatiotemporally Programmed Two‐Step Release Kinetics for Tumor Therapy
Source: Adv Sci (Weinh). 2019 Jun 14;6(16):1900586. doi: 10.1002/advs.201900586 (PMC6702759; doi:10.1002/advs.201900586)
Supplement: Supplementary file 1 — Supplementary [file ADVS-6-1900586-s001.pdf]

Copyright WILEY-VCH Verlag GmbH & Co. KGaA, 69469 Weinheim, Germany, 2019.

ADVANCED SCIENCE
Open Access

Supporting Information

for *Adv. Sci.,* DOI: 10.1002/advs.201900586

Thermoresponsive and Protease-Cleavable Interferon-Polypeptide Conjugates with Spatiotemporally Programmed Two-Step Release Kinetics for Tumor Therapy

*Zhuoran Wang, Jianwen Guo, Jiawei Sun, Ping Liang, Yan Wei, Xuliang Deng, and Weiping Gao**

Copyright WILEY-VCH Verlag GmbH & Co. KGaA, 69469 Weinheim, Germany, 2019.

Supporting Information

Thermoresponsive and Protease-Cleavable Interferon-Polypeptide Conjugates with Spatiotemporally-Programmed Two-Step Release Kinetics for Tumor Therapy

*Zhuoran Wang, Jianwen Guo, Jiawei Sun, Ping Liang, Yan Wei, Xuliang Deng, Weiping Gao**

Prof. W. Gao, Prof. X. Deng

Department of Geriatric Dentistry, Beijing Laboratory of Biomedical Materials, Peking University School and Hospital of Stomatology, Beijing, 100081, P.R. China

Biomedical Engineering Department, Peking University, Beijing, 100191, P.R. China

*Corresponding author. E-mail: gaoweiping@hsc.pku.edu.cn

Prof. Y. Wei

Department of Geriatric Dentistry, Beijing Laboratory of Biomedical Materials, Peking University School and Hospital of Stomatology, Beijing, 100081, P.R. China

Dr. Z. Wang, Dr. J. Guo, Dr. J. Sun

Department of Biomedical Engineering, School of Medicine, Tsinghua University, Beijing, 100084, P.R. China.

Dr. P. Liang

Department of Neurosurgery, Beijing Tsinghua Changgung Hospital, School of Clinical Medicine, Tsinghua University, Beijing, 102218, P.R. China.

Supplementary Materials and Methods

Materials

All molecular biology reagents were purchased from New England Biolabs unless otherwise specified. All chemical reagents were purchased from Sigma-Aldrich (St. Louis, MO) unless otherwise specified. All used antibiotics were acquired from BD Pharmingen (San Diego, CA, USA). All cell culture reagents and media were purchased from Gibco unless otherwise specified. Daudi B cells, human ovarian carcinoma OVCAR-3 cells and malignant melanoma C8161 cells were purchased from cell bank of Chinese Academy of Medical Sciences. Female BALB/c nude mice were purchased from Vital River Laboratories (Beijing, China) and accommodated in animal research facility of Tsinghua University, and it is accredited by the AAALAC (Association for Assessment and Accreditation of Laboratory Animal Care International). All animal procedures applied in this research are approved by the Institutional Animal Care and Use Committee (IACUC).

Construction, expression and purification of IFNα-MMPS-ELP(A), IFNα-ELP(A), IFNα-MMPS-ELP(V), IFNα-ELP(V) and IFNα

The gene encoding IFNα (NCBI GI 386795 for protein) was PCR-amplified from a previously synthesized IFN-containing pGEM®-T vector (Sangon Biotech, China) and inserted into a modified pET-24a (+) (Novagen Inc., Madison, WI) vector[1] using *BseR* I/*Acu* I restriction sites. The primers used are designed as follows:

Forward primer:

5’GAGATAGAGGAGTACATATGGGCTGTGATCTGCCTCAGACTCATT 3’

Reverse primer: 5’TTTCCGCTGAAGGCAGAGAGCCACCGCCACCGGATCCTTCTTTAGAACGCAGGCTCT 3’

The gene encoding MMP-2 substrate peptide (MMPS, GPLGLAGSRAGVG) was custom synthesized and inserted into the modified pET-24a (+) vector using *BseR* I/*Acu* I restriction sites. The primers used are designed as follows:

Forward primer: 5’GAGATAGAGGAGTACATATGGGCGGCCCGCTGGGTCTGGCAGGCA 3’

Reverse primer: 5'TTTCCGCTGAAGGCAGAGAGCCACCGCCAACTCCGGCACGGCTGC 3'

The vector encoding IFNα-MMPS was constructed by using the recursive directional ligation by plasmid reconstruction (PRe-RDL) method.[1] In brief, the IFNα-containing vector was digested with *BseR* I and *Bgl* I, and the MMPS-containing vector was digested with *Acu* I and *Bgl* I, and then the two compatible halves were ligated to reconstitute the original vector to contain IFNα-MMPS DNA fragment.

The DNA sequences of ELP(A) and ELP(V) (composed of 90 repeats of the pentapeptide Val-Pro-Gly-Val-Gly and Val-Pro-Gly-Ala-Gly, respectively) were inserted into the modified pET-24a (+) vector and added to the C-terminal of IFNα-MMPS or IFNα by using the PRe-RDL method as presented above to reconstitute the functional plasmids for IFNα-MMPS-ELP(A), IFNα-MMPS-ELP(V), IFNα-ELP(A) and IFNα-ELP(V).

After verified by DNA sequencing, the constructed plasmids encoding IFNα-MMPS-ELP(A), IFNα-MMPS-ELP(V), IFNα-ELP(A), IFNα-ELP(V) and IFNα were transformed into *E. coli* strain Rosetta-gami (DE3) pLysS competents (Invitrogen) and incubated in Luria Bertani medium containing 50 μg/mL ampicillin at 37 °C. The cultures were used to inoculate 1 L of sterile terrific broth medium with 250 rpm shaking until the optical density at 600 nm (OD_{600}) was 0.5, at which time the temperature was lowered to 25 °C and isopropyl-β-D-thiogalactopyranoside (IPTG) with a final concentration of 500 μM was added for expression induction overnight. Cells were collected and suspended in PBS solution, pH = 7.4 and lysed by sonication. The cell lysates were centrifuged. Nucleic acids in the extracts were precipitated by adding polyethyleneimine (1% w/v). After centrifugation, the pellets were discarded.

IFNα-MMPS-ELP(A), IFNα-MMPS-ELP(V), IFNα-ELP(A) and IFNα-ELP(V) in the supernatants were purified by inverse transition cycling (ITC), as described previously with minor modification.[2] The inverse phase transitions were initiated by the addition of NaCl to a final concentration of 3 M and the aggregated IFNα-MMPS-ELP and IFNα-ELP were separated from the solutions by centrifugation

at 16,000 × g at 37 °C for 15 min. The pellets were resuspended in cold PBS on ice for 15 min. The suspensions were centrifuged at 4 °C to remove any insoluble particles. This cycle was typically repeated 2 times. Purified IFNα-MMPS-ELP and IFNα-ELP were stored in PBS, pH = 7.4 at -80 °C for further use.

IFNα was purified by immobilized metal affinity chromatography as described in the published literature.[3] In brief, the supernatant was applied to a 5 mL HisTrap column (GE Healthcare) mounted in AKTA purifier system. The column was washed with equilibration buffer (50 mM Tris, 500 mM NaCl, 10% glycerol, 5 mM imidazole, pH 7.4) and then washing buffer (50 mM Tris, 500 mM NaCl, 10% glycerol, 50 mM imidazole, pH 7.4). His_6-tagged IFNα protein was finally eluted using the buffer containing 500 mM imidazole. The eluted IFNα was further purified on a HiPrep 26/10 desalting column (GE Healthcare) for buffer exchanging to 10 mM PBS, pH 7.4 and stored at -80 °C for further use.

The purification process was monitored by sodium dodecyl sulfate polyacrylamide gel electrophoresis (SDS-PAGE). The concentration of purified proteins was determined by bicinchoninic acid (BCA) assay according to the directions of BCA kit (Beyotime Biotech).

Physicochemical characterization

Matrix-assisted laser desorption/ionization time-of-flight mass spectrometry (MALDI-TOF-MS). The molecular weights of protein samples were analyzed with 4800 Plus MALDI-TOF/TOF™ Analyzer (Applied Biosystems). The matrix was a saturated solution of sinapic acid in a 1:1 mixture of acetonitrile and water containing 0.1% trifluoroacetic acid. The sample was diluted in water and then mixed with the matrix solution (1:1, v:v). The mixture of 1 µL was spotted onto a sample plate and dried in air at room temperature. The spectra were calibrated with proteins of known masses.

Dynamic light scattering (DLS). DLS was conducted with Zetasizer Nano-zs90 (Malvern) operating at a laser wavelength of 633 nm and a scattering angle of 90° at 4 °C for IFNα-MMPS-ELP(V), IFNα-

ELP(V) and 25 °C for IFNα-MMPS-ELP(A), IFNα-ELP(A) and IFNα. The samples were filtered (0.22 μm pore size, Millipore Corp.) before analysis. The data were analyzed with Zetasizer software 6.32. Particle size was expressed as intensity-weighted mean hydrodynamic radius.

Circular dichroism (CD). CD spectra were recorded in the range from 200 nm to 260 nm on Pistar π-180 (Applied Photophysics Ltd) instrument. The samples were diluted to a concentration of 0.15 mg/mL in H_2O.

Thermoresponsive phase transition. The phase transition behaviors of IFNα-MMPS-ELP(A), IFNα-MMPS-ELP(V), IFNα-ELP(A) and IFNα-ELP(V) were characterized by monitoring the absorbance at 350 nm as a function of temperature on SpectraMax M3 Microplate Reader (Molecular Devices) by heating an fusion protein sample solution in PBS, typically from 4 °C to 80 °C at a rate of 1 °C/min. The transition temperature (T_t) was defined from the heating profile as the temperature at 50% of maximum turbidity. To study the T_t as a function of concentration for IFNα-MMPS-ELP, disposable cuvettes containing 200 μL samples at different concentrations were incubated from 4 to 80 °C, and the absorbance at 350 nm of the sample was monitored every 2 °C.

Cleavage of IFNα-MMPS-ELP with MMP-2 in buffer

Human MMP-2 enzyme was first activated with p-aminophenylmercuric acetate (APMA) according to the instructions as follows: After dilution to 100 μg/mL in the assay buffer (50 mM Tris, 10 mM $CaCl_2$, 150 mM NaCl, and 0.05% (w/v) Brij 35, pH 7.5), MMP-2 was activated by adding APMA to a final concentration of 1 mM and incubated at 37 °C for 1 h. Then, 100 μg of IFNα-MMPS-ELP or IFNα-ELP protein samples were added into 500 μL of the digestion system. The mixture was incubated for another 6 h at 37 °C. The incubation products were monitored by SDS-PAGE.

Cell culture

Human ovarian carcinoma OVCAR-3 cells, malignant melanoma C8161 cells and Daudi B cells were cultured in 25 cm^2 flasks and maintained in a humidified 5% CO_2 incubator at 37 °C, with use of

Dulbecco's Modified Eagle Medium (DMEM, Gibco, USA) containing 100 U/ml penicillin, 100 mg/ml streptomycin and 10% FBS (Gibco, USA).

Cleavage of IFNα-MMPS-ELP with MMP-2 in tumor cell-conditioned media

OVCAR-3 and C8161 tumor cells were seeded into a 6-cm dish with a density of 5 × 10^6 cells. After 4 h incubation, cells adhered to the dish, and then the culture medium was replaced with fresh medium without FBS. After 24 h incubation, the medium was collected and centrifuged at 12,000 rpm for 10 min at 4 °C to remove the cell debris. IFNα-MMPS-ELP or IFNα-ELP was incubated with the OVCAR-3 and C8161 tumor cells conditioned media at 37 °C for 48 h. The cleavage efficiency of IFNα-MMPS-ELP or IFNα-ELP by MMP-2 in tumor cell-conditioned media was detected by using SDS-PAGE.

***In vitro* antiproliferative activity**

Daudi B cells were cultured in DMEM medium containing 10% (vol/vol) fetal bovine serum (FBS) and 1% penicillin/streptomycin (Hyclone) at 37 °C in a humidified, 5% CO_2 atmosphere. Cells were seeded at a density of 5,000 per well in a 96-well plate (Corning) and serial dilutions (1, 2, 5, 10, 20, 50, 100, 300, 1000, 10000 pg/mL) of the IFNα, IFNα-ELP(A), IFNα-ELP(V), IFNα-MMPS-ELP(A) and IFNα-MMPS-ELP(V) samples in fresh medium were added. Wells filled with media were defined as 0% viability. Wells filled with PBS-treated cells only were defined as 100% viability. After incubating the plate for 96 h, the proliferation of cells was determined by MTT assay according to Cell Proliferation Assay kit (Promega). The data fitting and IC_{50} calculation were analyzed by GraphPad Prism 5.0 software and presented as mean ± standard deviation.

After the IFNα-MMPS-ELP or IFNα-ELP samples were cleaved by protease MMP-2 *in vitro* as described above, serial dilutions of the cleavage product samples in fresh medium were added to Daudi B cells culture. The proliferation of cells was detected by MTT assay as mentioned above.

Tumor penetration of IFNα-MMPS-ELP

The efficiency of IFNα-MMPS-ELP(A) to penetrate in tumor was examined in nude mice bear C8161 tumors. IFNα, IFNα-ELP(A) and IFNα-MMPS-ELP(A) were labelled with Cy5 (lumiprobe) dye according to manufacturer's protocol. When the tumor volume reached 50 mm^3, saline and Cy5-tagged IFNα, IFNα-ELP(A) and IFNα-MMPS-ELP(A) were intravenously injected to the mice at the same dose of 5 mg IFN-equivalent/kg body weighy (BW). 8 h later, the tumor tissues were collected, embedded, and cryosectioned to 8 μm thickness slides. The tissues were fixed with paraformaldehyde solution (4% wt/vol) for 15 min, followed by blocking with 5% (wt/vol) BSA at 37℃ for 1 h. Tumor sections were stained for vasculature using anti-CD31 (cell adhesion molecule on endothelial cells) as primary antibody, followed by incubation with a goat anti-mouse Cy3-conjugated IgG secondary antibody. Then the nucleus was stained with DAPI. The sections were then washed, covered with coverslip. Then the Cy5 fluorescence intensity was observed and images were achieved with a LSM710 laser scanning confocal microscope at the emission and excitation wavelengths of 670 nm and 650 nm. In addition, the Cy5 fluorescence intensity of the images was calculated with Image J software for comparison.

To evaluate the penetration efficiency of IFNα-MMPS-ELP(V) in tumor, saline and Cy5-tagged IFNα, IFNα-ELP(V), IFNα-MMPS-ELP(A) and IFNα-MMPS-ELP(V) were subcutaneously injected to the mice at their MTDs. 12 h later, the tumor tissues were collected and treated for fluorescence imaging as mentioned above.

Maximum tolerated dose (MTD)

Healthy female SPF BALB/c mice (6 weeks old, 18 g) were administered subcutaneously with IFNα-MMPS-ELP(V) and IFNα-ELP(V) at the doses of 25, 50, 100, 200 mg IFNα-equivalent/kg BW, IFNα-MMPS-ELP(A) at the doses of 10, 15, 20, 25 mg IFNα-equivalent/kg BW or IFN at the doses of 5, 10, 15, 20 mg IFNα/kg BW (n = 3 for each injection). Mouse survival and body weight change were monitored daily for two weeks. Mice that lost > 10% of their pre-treatment body weights were

euthanized. The highest dose at which no animal mortality and no more than 10% body weight loss were observed was defined as the MTD.

In addition, saline and Cy5-tagged IFNα, IFNα-ELP(V), IFNα-MMPS-ELP(A) and IFNα-MMPS-ELP(V) were subcutaneously injected to the mice at their MTDs. *In vivo* fluorescence imaging was performed at the indicated time points (0, 1, 2, 3, 5, 10, 20, 30, 40 d) to evaluate the release of conjugates under skin during 40 days.

Pharmacokinetics

Healthy female SPF BALB/c nude mice with an average body weight of about 20 g were used to evaluate the pharmacokinetics of IFNα-MMPS-ELP(A) compared with IFNα-ELP(A) and IFNα. The mice were randomly distributed to 3 groups (n = 3 for each group), and each group received an intravenous injection of IFNα, IFNα-ELP(A) or IFNα-MMPS-ELP(A) at the same dose of 1 mg IFNα-equivalent/kg BW. At selected time points (1, 5, 15, 30 min, 1, 2, 8, 24, 48, 72 h), blood samples (100 μL) were drawn from retro orbital after anesthesia with isoflurane, centrifuged at 4,000 × g for 15 min after standing for 30 min at 4 ℃. The plasma was collected and stored at -80 ℃ for further use. The concentration of IFNα equivalent was determined by ELISA assay according to the instruction of human IFN-α2 ELISA kit (PBL Interferon Source). Simultaneously, the plasma from untreated mice (control) was obtained and defined as the background. The data were fitted with a two-compartment model by using Drug analysis System 3.0 (DAS 3.0) software package (Mathematical Pharmacology Professional Committee of China, Shanghai, China) to produce pharmacokinetic parameters.

To study the pharmacokinetics of IFNα-MMPS-ELP(V), the mice in each group (n = 3) were subcutaneously injected with IFNα-MMPS-ELP(V), IFNα-ELP(V), IFNα-MMPS-ELP(A) or IFNα at its MTD. At selected time points (1, 5, 15, 30 min, 1, 2, 8 h, 1, 2, 3, 5, 7 d, and then once every 3 days), blood samples (100 μL) were harvested and centrifuged at 4,000 × g for 15 min after standing for 30 min at 4 ℃. The plasma was collected and stored at -80 ℃ for further use. The methods of

measuring IFNα concentrations were the same as above. The data were fitted with a one-compartment model by using DAS 3.0 software to produce pharmacokinetic parameters.

Biodistribution

The human malignant melanoma C8161 cell line was grown in DMEM Medium containing 10% fetal bovine serum (FBS) and 1% penicillin/streptomycin. Female BALB/c nude mice of 6 weeks old were subcutaneously inoculated in the right dorsal area with 5×10^6 C8161 cells (0.2 mL) suspended in DMEM medium. When the tumors grew to a size of 100-150 mm^3 (~ 6 weeks), the mice were randomly grouped into 3 groups (3 mice in each group) and received intravenous injection of IFNα, IFNα-ELP(A) and IFNα-MMPS-ELP(A) at the same dose of 1.5 mg IFNα-equivalent/kg BW. At 8 h after the injections, the mice were sacrificed by CO_2 and major tissues (tumor, heart, kidney, liver, spleen, lung, pancreas, stomach, muscles and intestine) were harvested. The collected tissues were then weighed, homogenized and suspended in the corresponding quantity of 10 mM PBS extraction buffer containing 1 mM EDTA, 1% Triton X-100, 0.5% sodium deoxycholate, 1 mM PMSF, phosphatase inhibitor cocktail 2 and 3 (1:100 diluted) and protease inhibitor cocktail (1:100 diluted). The concentrations of IFNα equivalent in the samples were quantified by ELISA as described above. The background of tissues from untreated mice was subtracted from the acquired data correspondingly. The data were presented as IFNα equivalent (ng) per gram of tissue (ng/g tissue).

To study the biodistribution of IFNα-MMPS-ELP(V), the mice in each group (n = 3) were subcutaneously injected with IFNα, IFNα-ELP(V), IFNα-MMPS-ELP(A) and IFNα-MMPS-ELP(V) at their MTDs, respectively. At 24 h, 72 h and 30 d after the injections, the mice were sacrificed by CO_2 and major tissues were harvested. The concentrations of IFNα equivalent in the samples were quantified by ELISA as described above.

Antitumor efficacy

C8161 melanoma cells were cultured in DMEM medium containing 10% (vol/vol) FBS and 1% penicillin/streptomycin at 37 $^{\circ}C$ in a humidified, 5% CO_2 atmosphere. Female BALB/c nude mice of 6

weeks old were subcutaneously inoculated in the right dorsal area with 5 × 10^6 C8161 cells (0.1 mL) suspended in DMEM medium. At 15 days post the inoculations, the tumors got established with an average size of ~30 mm^3. The animals were randomized to 4 groups (n = 8 to 10 per group) and acquired intravenous injections of IFNα-MMPS-ELP(A), IFNα-ELP(A), and IFNα at the same dose of 1.5 mg IFNα-equivalent/kg BW and saline at the equivalent volume (100 μL) every 3 days for 10 times, respectively.

To evaluate the antitumor efficacy of IFNα-MMPS-ELP(V), C8161 melanoma cells and OVCAR-3 ovarian cells were cultured in DMEM medium containing 10% (vol/vol) FBS and 1% penicillin/streptomycin at 37 °C in a humidified, 5% CO_2 atmosphere. Female BALB/c nude mice of 6 weeks old were subcutaneously inoculated in the right dorsal area with 5 × 10^6 C8161 cells or OVCAR-3 cells (0.1 mL) suspended in DMEM medium. At 15 days and 40 days post the inoculations, the two kinds of tumors got established with average sizes of ~30 and ~60 mm^3, respectively. The animals were randomized to 5 groups (n = 8 to 10 per group for melanoma and n = 6 to 8 per group for ovarian tumors) and acquired subcutaneous injections of IFNα-MMPS-ELP(V), IFNα-ELP(V), IFNα-MMPS-ELP(A) and IFNα at their MTDs, and saline at the equivalent volume (100 μL), respectively.

Tumor sizes were measured via calipers every 3 days and calculated using the formula: volume = length × $width^2$ × 0.5. Simultaneously, the mice were weighed. The mice would be considered dead if their tumor volumes were larger than 300 mm^3 or the body weight loss was greater than 15%.

Biological safety

To evaluate the *in vivo* safety of IFNα, IFNα-ELP(V), IFNα-MMPS-ELP(A) and IFNα-MMPS-ELP(V), melanoma-bearing mice were sacrificed on the 12th day post subcutaneous injections at their MTDs, tissues including tumor, heart, kidney, spleen, lung and liver were collected. The harvested tissues were fixed with formalin, and embedded in paraffin for histological examination. Tissue sections (5 µm in thickness) mounted onto glass slides were stained with hematoxylin-eosin (H&E) for

morphology observation according to standard procedures. The images of all sections were captured with Nikon Eclipse 90i microscopy.

At the end of the treatments, blood was collected via retro orbital for hematology examination. Hematological parameters of complete blood were measured with Hematology Analyzer (SYSMEX), including number counts of white blood cells (WBC), red blood cells (RBC), platelets (PLT) and the concentration of hemoglobin (HGB). Clinical biochemistry parameters of serum were measured by Automatic Biochemical Analyzer (HITACHI), including lactate dehydrogenase (LDH) and creatine kinase isoenzymes (CK-MB) which are markers for heart function, aspartate aminotransferase (AST) and alanine aminotransferase (ALT) which are markers for hepatic function, creatinine (CREA) and blood urea nitrogen (UREA) which are markers for kidney function.

Statistical analysis

Data were evaluated as the mean ± standard error of the mean. For antitumor study, values were analyzed with variance (ANOVA) test and then Tukey HSD test for multiple comparisons between different groups by SPSS software (SPSS, Chicago IL, USA). The value of $P < 0.05$ was considered to represent statistical significance. Statistical analyses were performed with GraphPad Prism 5.0 software (Graphpad Software Inc., La Jolla, California).

Supplementary Figures and Tables

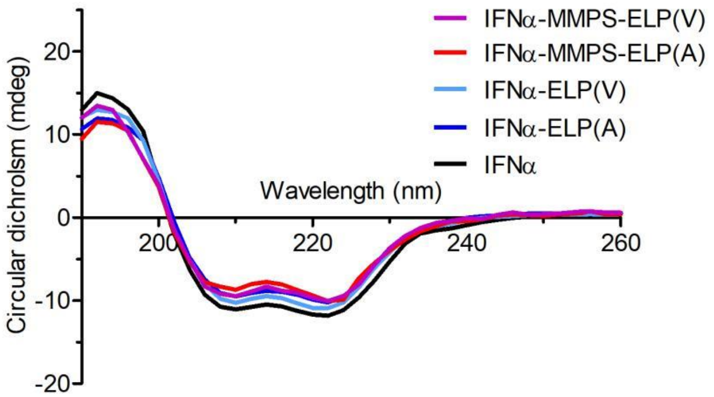

Figure S1. CD spectra of IFNα, IFNα-ELP(A), IFNα-ELP(V), IFNα-MMPS-ELP(A) and IFNα-MMPS-ELP(V).

The circular dichroism (CD) spectra showed that IFNα exhibited two negative minima at 209 and 221 nm characteristic of an α-helix structure of protein. The CD shape and the band intensity of IFNα-ELP(A), IFNα-ELP(V), IFNα-MMPS-ELP(A) and IFNα-MMPS-ELP(V) were close to that of IFNα, indicating that the secondary structure of IFNα did not change after the ELP conjugation.

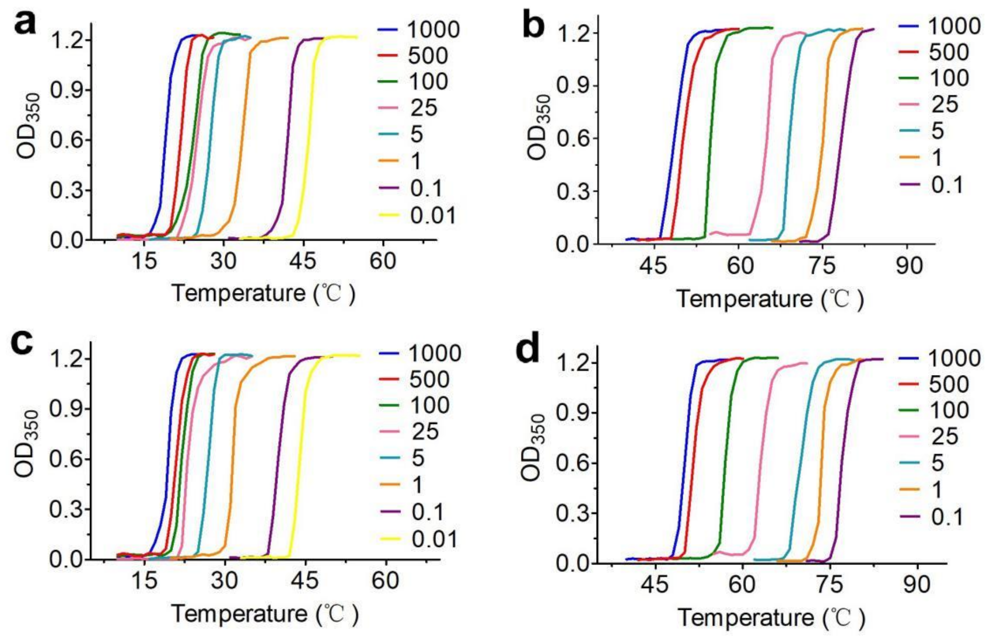

Figure S2. Turbidity profiles of IFNα-MMPS-ELP(V) (a), IFNα-MMPS-ELP(A) (b), IFNα-ELP(V) (c) and IFNα-ELP(A) (d) as a function of temperature at different concentrations.

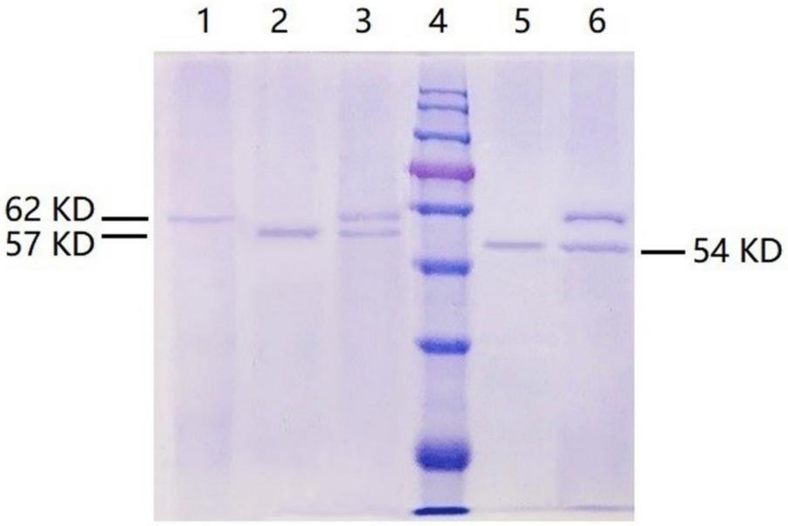

Figure S3. SDS-PAGE analyses of IFNα-ELP(V) and IFNα-ELP(A) after incubation with MMP-2. Lane 1: MMP-2 (62 kD), lane 2: IFNα-ELP(V) (57 kD), lane 3: IFNα-ELP(V) incubated with MMP-2 (MMP-2 62 KD, IFNα-ELP(V) 57 KD), lane 4: protein marker, lane 5: IFNα-ELP(A) (54 kD), lane 6: IFNα-ELP(A) incubated with MMP-2 (MMP-2 62 KD, IFNα-ELP(A) 54 KD).

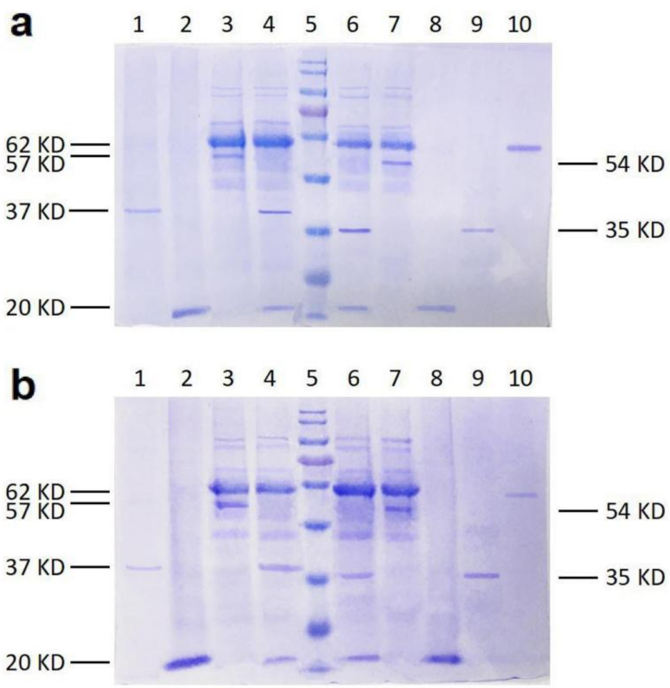

Figure S4. SDS-PAGE analyses of IFNα-ELP(A), IFNα-ELP(V), IFNα-MMPS-ELP(A) and IFNα-MMPS-ELP(V) incubated with ovarian (a) and melanoma (b) cell-conditioned media for 48 h. Lane 1: ELP(V) (37 KD), lane 2 and 8: IFNα (20 KD), lane 3: IFNα-ELP(V) incubated with tumor cell-conditioned media (MMP-2 62 KD, IFNα-ELP(V) 57 KD), lane 4: IFNα-MMPS-ELP(V) incubated with tumor cell-conditioned media (MMP-2 62 KD, ELP(V) 37 KD, IFNα 20 KD), lane 5: protein marker, lane 6: IFNα-MMPS-ELP(A) incubated with tumor cell-conditioned media (MMP-2 62 KD, ELP(A) 35 KD, IFNα 20 KD), lane 7: IFNα-ELP(A) incubated with tumor cell-conditioned media (MMP-2 62 KD, IFNα-ELP(A) 54 KD), lane 9: ELP(A) (35 KD), lane 10: MMP-2 (62 KD).

After incubated with ovarian (a) and melanoma (b) cell-conditioned media for 48 h, IFNα-MMPS-ELP(V) and IFNα-MMPS-ELP(A) were cleaved into two species of IFNα and ELP, while IFNα-ELP(V) and IFNα-ELP(A) could not be cleaved.

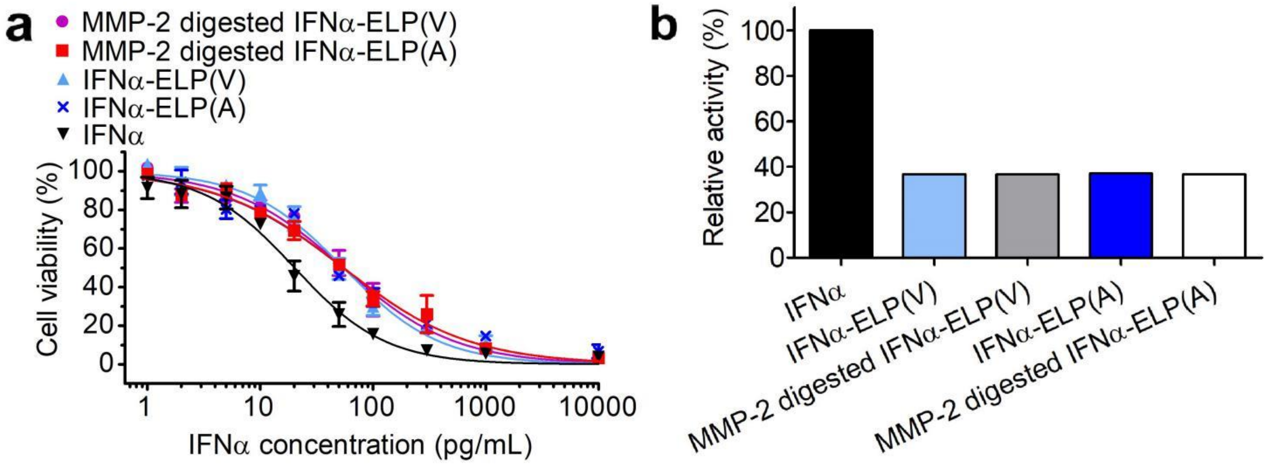

Figure S5. *In vitro* cytotoxicity of IFNα-ELP(V) and IFNα-ELP(A) to Daudi B cells after MMP-2 treatments (a) and the relative antiproliferative activities after MMP-2 treatments as compared to that of IFNα (b).

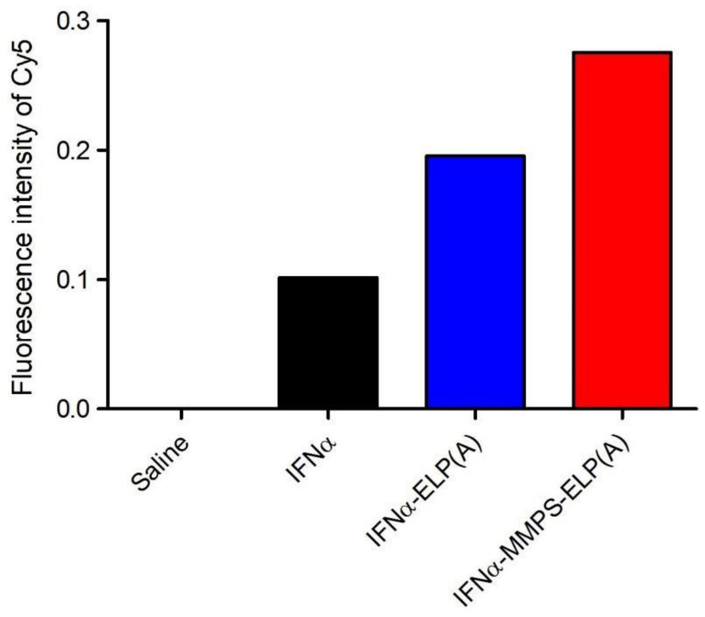

Figure S6. The quantitative analysis of Cy5 fluorescence intensity in Fig. 2c.

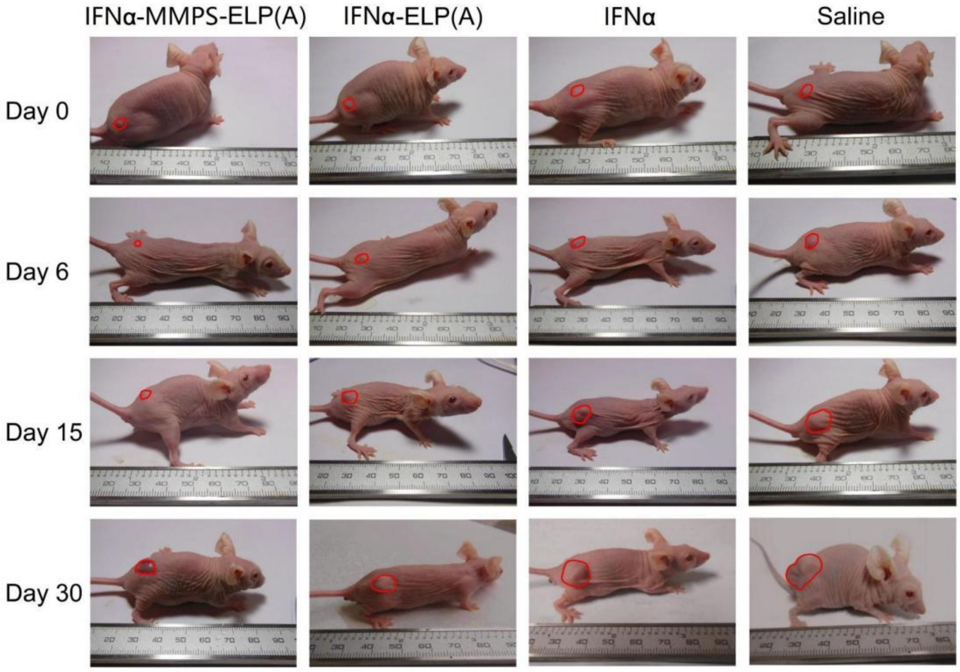

Figure S7. Representative images for melanoma growth post intravenous injections of IFNα-MMPS-ELP(A), IFNα-ELP(A) and IFNα at the same dose of 1.5 mg IFNα-equivalent/kg BW.

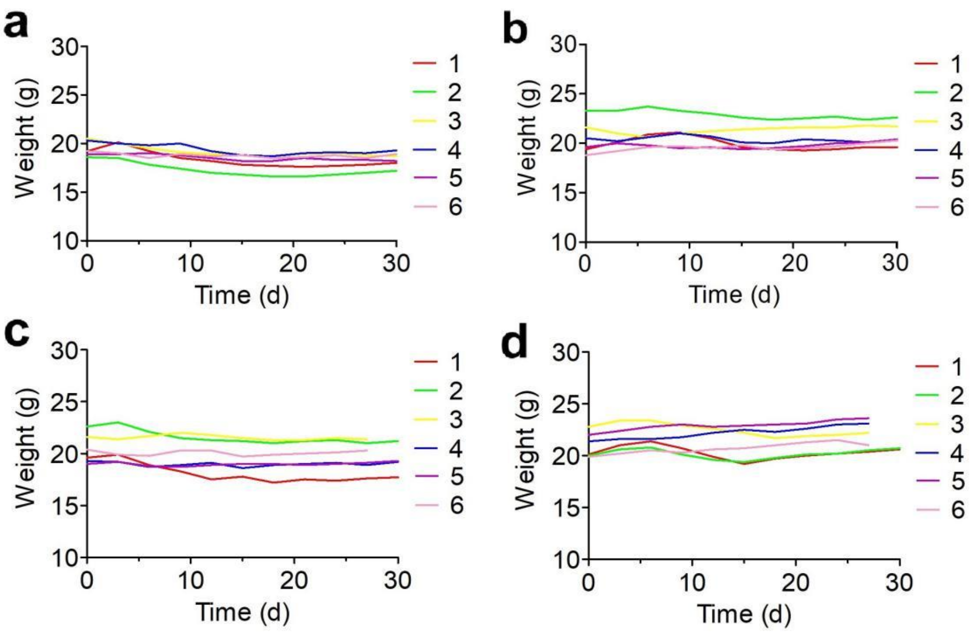

Figure S8. The change of mouse body weight after intravenous injections of IFNα-MMPS-ELP(A) (a), IFNα-ELP(A) (b), IFNα (c) and saline (d) at the same dose of 1.5 mg IFNα-equivalent/kg BW.

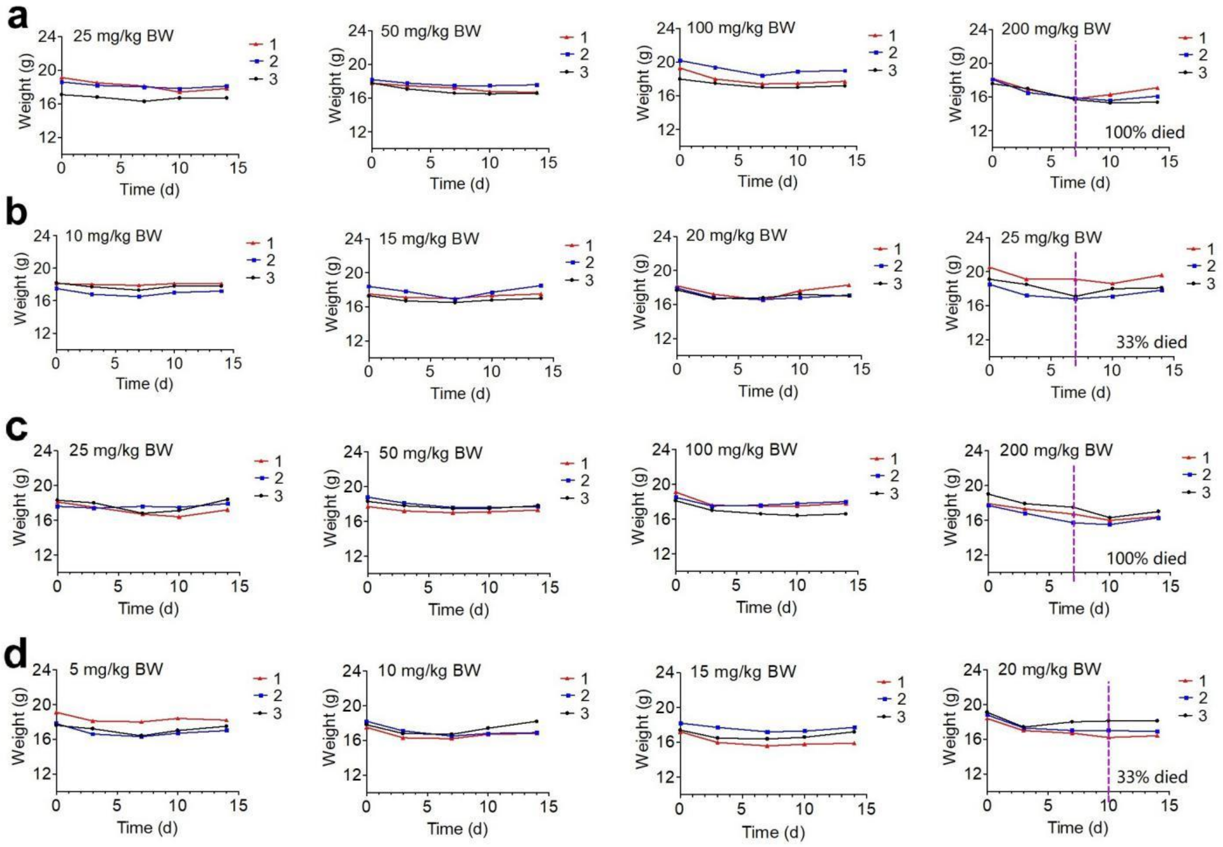

Figure S9. Dose escalation of IFNα-MMPS-ELP(V) (a), IFNα-MMPS-ELP(A) (b), IFNα-ELP(V) (c) and IFNα (d) in mice (n = 3). The vertical dash lines denote the time points at which more than 10% body weight loss was observed.

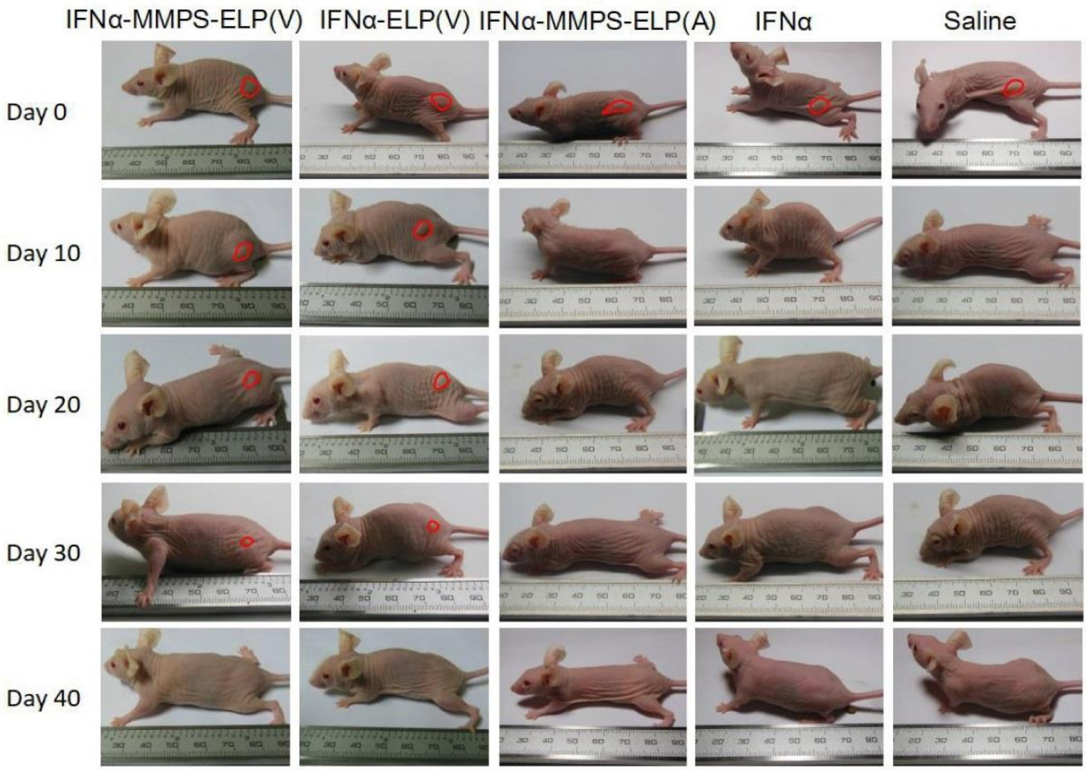

Figure S10. Representative images for IFNα-MMPS-ELP(V) and IFNα-ELP(V) depots at given time points post subcutaneous injections at their MTDs.

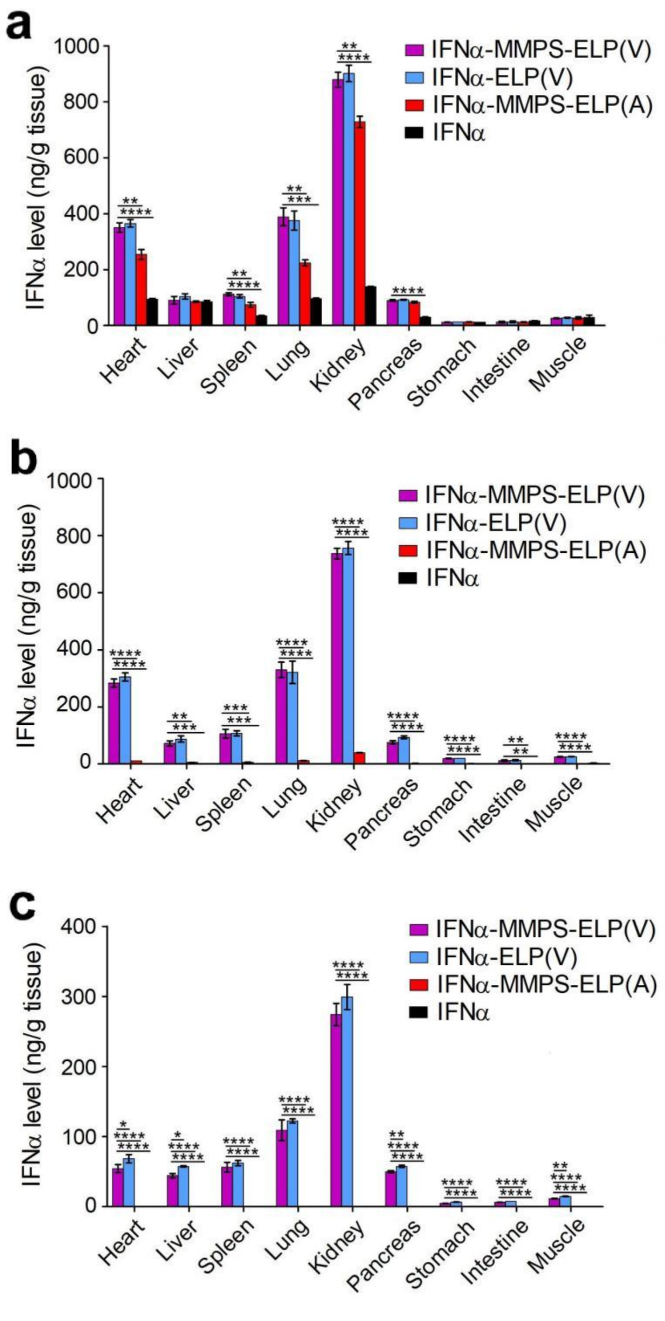

Figure S11. Biodistribution of IFNα-MMPS-ELP(V), IFNα-MMPS-ELP(A), IFNα-ELP(V) and IFNα at 1 d (a), 3 d (b) and 30 d (c) post administrations at their MTDs in a melanoma mouse model (n = 3). *P < 0.05, **P < 0.01, ***P < 0.001, ****P < 0.0001, significant difference for IFNα-MMPS-ELP(V) compared with IFNα-MMPS-ELP(A), IFNα-ELP(V) and IFNα. Data are shown as mean ± standard error of the mean.

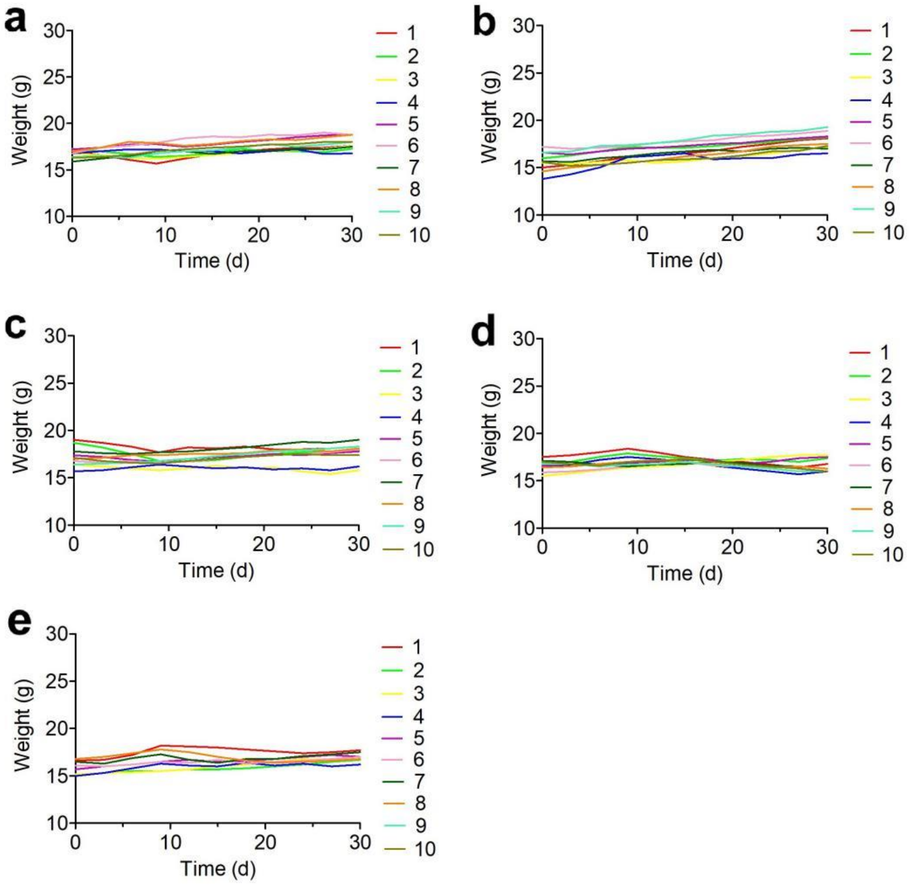

Figure S12. The change of mouse body weight after subcutaneous injections of IFNα-MMPS-ELP(V) (a), IFNα-MMPS-ELP(A) (b), IFNα-ELP(V) (c), IFNα (d) and saline (e) at their MTDs in a melanoma mouse model.

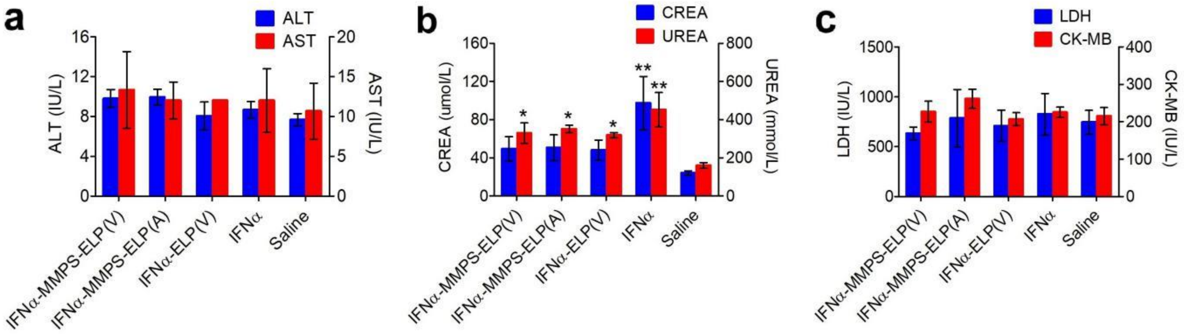

Figure S13. Biochemistry examinations for mice at 60 d after subcutaneous injections of IFNα-MMPS-ELP(V), IFNα-MMPS-ELP(A), IFNα-ELP(V) and IFNα at their MTDs in a melanoma mouse model. (a) Liver function markers: ALT, alanine aminotransferase; AST, aspartate aminotransferase. (b) Kidney function markers: CREA, creatinine; UREA, blood urea nitrogen. (c) Heart function markers: LDH, lactate dehydrogenase; CK-MB, creatine kinase isoenzymes. In all the experiments, n = 3, **P* < 0.05, ***P* < 0.01, significant difference for IFNα-MMPS-ELP(V), IFNα-MMPS-ELP(A), IFNα-ELP(V) and IFNα relative to saline. In panel b, the levels of CREA and UREA for IFNα are much higher than those for saline, suggesting the kidney damage caused by IFNα.

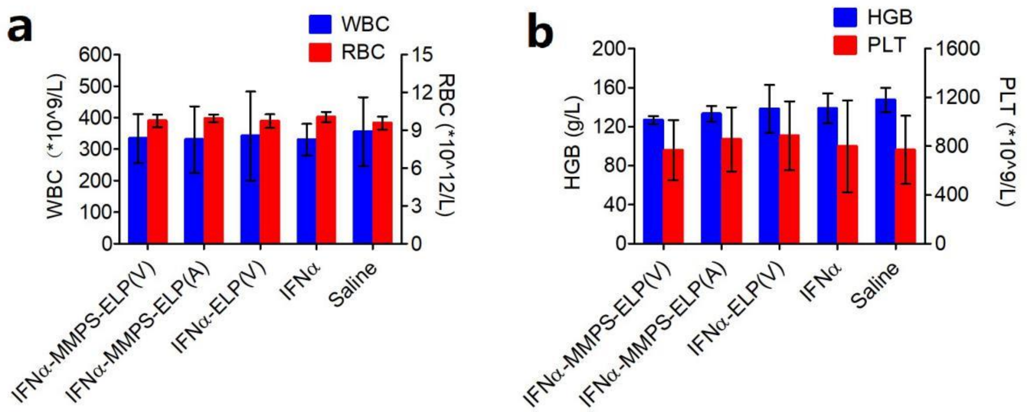

Figure S14. Hematological examinations for mice at 60 d after subcutaneous injections of IFNα-MMPS-ELP(V), IFNα-MMPS-ELP(A), IFNα-ELP(V) and IFNα at their MTDs in a melanoma mouse model. (a) WBC, white blood cells; RBC, red blood cells. (b) PLT, platelets; HGB, hemoglobin.

Table S1. Pharmacokinetic parameters of IFNα-MMPS-ELP(A), IFNα-ELP(A) and IFNα after intravenous injections at the same dose of 1 mg IFN-equivalent/kg BW (n = 3).

Parameter	IFNα	IFNα-ELP(A)	IFNα-MMPS-ELP(A)
Terminal half-life $T_{1/2\beta}$ (h)	1.4 ± 0.21	9.6 ± 2.7	8.9 ± 1.0
Area under curve AUC (μg/L · h)	61.8 ± 1.7	707.3 ± 37.1	684.1 ± 12.1
MRT (h)	1.3 ± 0.093	11.8 ± 0.84	10.8 ± 1.4
T_{max} (h)	0.017 ± 0	0.017 ± 0	0.017 ± 0
C_{max} (μg/L)	121.3 ± 0.045	131.5± 0.5	106.1 ± 4.8
Elimination rate constant Ke (1/h)	2.0 ± 0.072	0.19 ± 0.01	0.21± 0.06

Note: The data are presented as IFNα-equivalent.

Table S2. Pharmacokinetic parameters of IFNα-MMPS-ELP(V), IFNα-MMPS-ELP(A), IFNα-ELP(V) and IFNα after subcutaneous injections at their MTDs (n = 3).

Parameter	IFNα	IFNα-ELP(V)	IFNα-MMPS-ELP(A)	IFNα-MMPS-ELP(V)
Half-life time $T_{1/2}$ (h)	1.9 ± 0.08	491.8± 38.1 (21 d)	9.0 ± 0.87	422.2 ± 13.7 (18 d)
Area under curve AUC (mg/L·h)	46.9 ± 7.8	3102.0 ± 269.8	118.3 ± 15.1	2755.9 ± 16.8
MRT (h)	5.6 ± 0.64	380.1 ± 25.2	13.7 ± 0.4	346.4 ± 7.9
T_{max} (h)	2.7 ± 1.2	13.3 ± 9.2	4 ± 0	13.3 ± 9.2
C_{max} (μg/L)	5456.9 ± 758.5	5461.1 ± 558.8	5878.7 ± 187.1	5416.9 ± 589.3
Elimination rate constant Ke (1/h)	0.36 ± 0.02	0.0013 ± 0.0006	0.078 ± 0.008	0.002 ± 0

Note: The data are presented as IFNα-equivalent.

References

[1] J. R. McDaniel, J. A. MacKay, F. C. Quiroz, A. Chilkoti, *Biomacromolecules* **2010**, *11*, 944.

[2] Z. Wang, Q. He, W. Zhao, J. Luo, W. Gao, *J. Control. Release* **2017**, *264*, 66.
